# Signaling pathway mechanisms of circadian clock gene Bmal1 regulating bone and cartilage metabolism: a review

**DOI:** 10.1038/s41413-025-00403-6

**Published:** 2025-01-27

**Authors:** Yiting Ze, Yongyao Wu, Zhen Tan, Rui Li, Rong Li, Wenzhen Gao, Qing Zhao

**Affiliations:** 1 Department of Orthodontics, State Key Laboratory of Oral Diseases, National Clinical Research Center for Oral Diseases, West China Hospital of Stomatology, Sichuan University, Chengdu, Sichuan China; 2 Department of Implant Dentistry, State Key Laboratory of Oral Diseases, National Clinical Research Center for Oral Diseases, West China Hospital of Stomatology, Sichuan University, Chengdu, Sichuan China

**Keywords:** Metabolism, Metabolic disorders

## Abstract

Circadian rhythm is ubiquitous in nature. Circadian clock genes such as *Bmal1* and *Clock* form a multi-level transcription-translation feedback network, and regulate a variety of physiological and pathological processes, including bone and cartilage metabolism. Deletion of the core clock gene *Bmal1* leads to pathological bone alterations, while the phenotypes are not consistent. Studies have shown that multiple signaling pathways are involved in the process of *Bmal1* regulating bone and cartilage metabolism, but the exact regulatory mechanisms remain unclear. This paper reviews the signaling pathways by which *Bmal1* regulates bone/cartilage metabolism, the upstream regulatory factors that control *Bmal1*, and the current *Bmal1* knockout mouse models for research. We hope to provide new insights for the prevention and treatment of bone/cartilage diseases related to circadian rhythms.

## Introduction

To adapt to the environmental changes caused by the Earth’s rotation, mammals have developed a circadian biological clock with a period of approximately 24 h.^1^ Disruption to the circadian rhythms can lead to many suboptimal health conditions and diseases, such as obesity, diabetes, osteoarthritis, and cardiovascular diseases.^2–5^ The circadian rhythm biological clock consists of a central oscillator and peripheral oscillators. The central oscillator is located in the suprachiasmatic nucleus (SCN) of the hypothalamus, which receives light signals of external light-dark cycles through the retina, and then transmits them to peripheral oscillators through neural networks and hormonal signals.^6,7^ Clock genes, also known as “circadian clock genes,” are the molecular basis of rhythm expression. *Brain and muscle arnt-like 1* (*Bmal1*) is the core gene responsible for maintaining the rhythm, expressed in both the SCN and peripheral tissues such as bone and stem cells.^8,9^ CLOCK (Circadian locomotor output cycles kaput, CLOCK) can bind with BMAL1 to form heterodimers, which then bind and activate the transcription of clock-controlled genes (CCGs) containing E-box elements.^10,11^ *Period* (*Per*) and *Cryptochrome* (*Cry*) are also primary clock genes, and their transcription is initiated by the BMAL1/CLOCK heterodimers. Additionally, PER and CRY can inhibit the transcription of *Bmal1* and *Clock*, thus forming a negative feedback loop.^8,12,13^ Furthermore, nuclear transcription factor retinoid acid receptor related orphan receptor α (RORα) and nuclear receptor Rev-erbα can enhance and inhibit *Bmal1* transcription, respectively.^14^

The skeleton consists of bone and cartilage, which are respectively generated by osteoblasts and chondrocytes.^15^ Skeletal stem and progenitor cells are multipotent cells that can differentiate into osteoblasts, chondrocytes, adipocytes, and other lineages.^16–18^ During embryonic development, the skeleton forms through two classic processes: intramembranous ossification and endochondral ossification.^19^ For example, in the craniofacial skeleton, bone primarily forms through intramembranous ossification. Mesenchymal cells differentiate into osteoblasts, deposit a matrix rich in type I collagen, and eventually differentiated osteocytes embed into the bone matrix, forming bone.^20^ Long bones and axial skeleton are formed via endochondral ossification, where mesenchymal precursors condense to form hypertrophic chondrocytes, producing avascular cartilaginous matrix rich in type X collagen. Subsequently, hypertrophic chondrocytes in the central region initiate remodeling by producing specific matrix metalloproteinases (MMPs), mineralizing the hypertrophic cartilage directly, and attracting vascularization through the release of vascular endothelial growth factor (VEGF).^21^

*Bmal1*, as the core clock gene, is expressed in a widespread manner from mesenchymal stem cells (MSCs) to the terminal differentiated osteocytes and chondrocytes, and numerous studies have shown that bone and cartilage metabolism exhibit diurnal rhythmic characteristics, with most bone metabolism markers showing diurnal oscillations, typically presenting as nighttime peaks and daytime troughs.^22^ Furthermore, it has been demonstrated that *Bmal1* can affect many crucial factors for skeletal development, such as the transcription factors runt-related transcription factor 2 (RUNX2), SRY-related high mobility group-box 9 (SOX9), bone morphogenetic protein (BMP), Wnt signaling pathway, etc.^23–27^ Deletion of *Bmal1* in mice results in pathological bone alterations. The importance of *Bmal1* in the metabolism of bone and cartilage is evident. Therefore, this review systematically describes recent research on the signaling pathways involved in *Bmal1* regulating bone/cartilage metabolism, the upstream agents that modulate *Bmal1* expression, and the various *Bmal1* knockout mouse models to provide a deeper understanding of physiological bone/cartilage metabolism, as well as *Bmal1*’s potential value for treating bone/cartilage diseases.

## *Bmal1* affects bone/cartilage metabolism

**Fig. 1:**
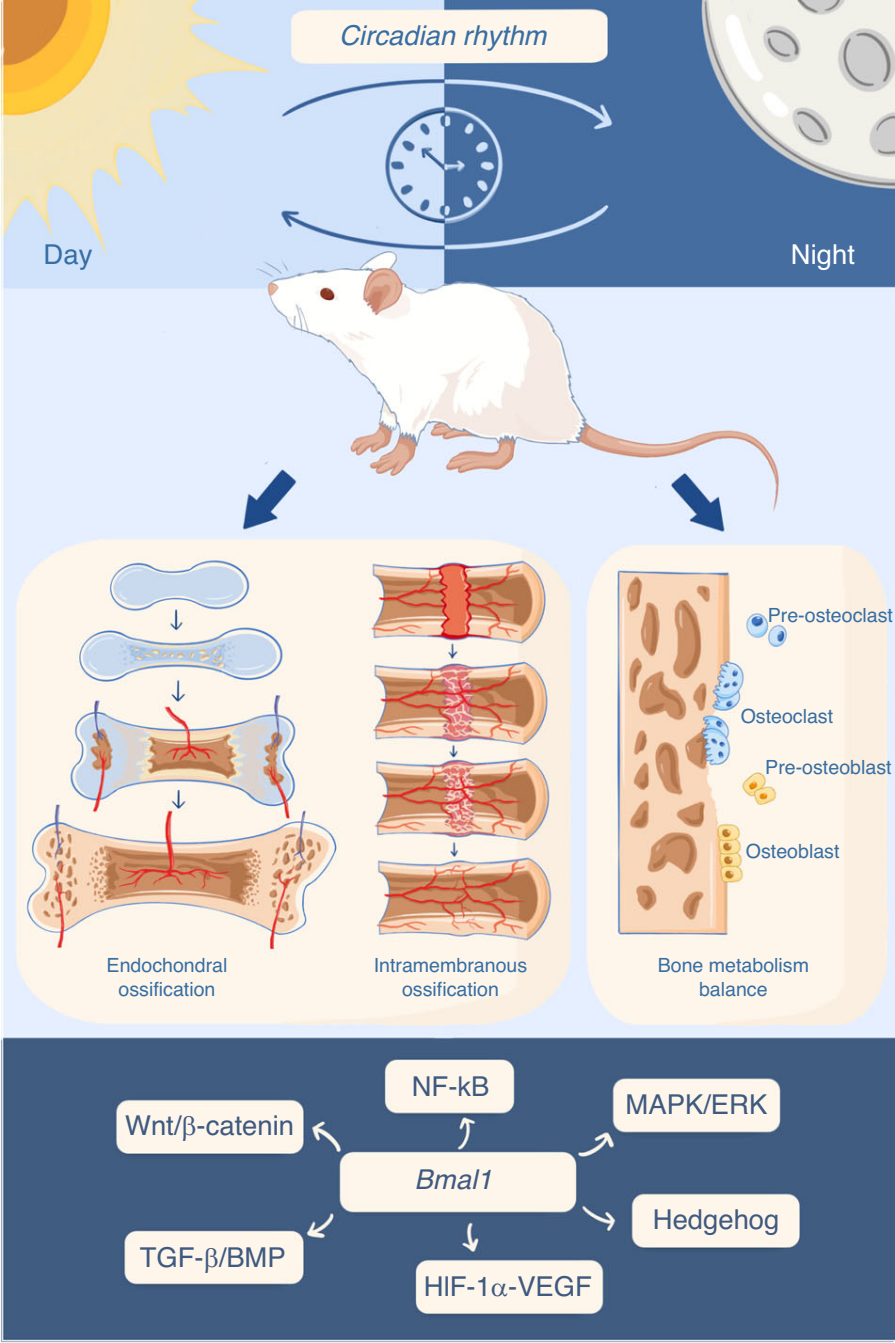
Circadian rhythm influences bone/cartilage metabolism. Endochondral ossification involves the formation of cartilage template, differentiation and proliferation of chondrocytes, and gradual replacement by osteoblasts, resulting in bone formation. Intramembranous ossification entails the gradual differentiation of MSCs within soft tissues (or damaged tissues) into osteoblasts, followed by the formation of primary bone spicules and gradual deposition of mature bone tissue. Under physiological conditions, bone formation and resorption are dynamically balanced. Clock genes have been implicated in the regulation of the aforementioned bone/cartilage metabolism processes through various signaling pathways or networks

Bone and cartilage metabolism is a complex biological process, involving the dynamic balance of bone formation and bone resorption, as well as specific cells such as bone marrow mesenchymal stem cells (BMSCs), osteoblasts, osteoclasts, and chondrocytes.^28^ Osteoclasts dissolve the inorganic salts and organic matrix of bone tissue by releasing acidic protons and enzymatic molecules such as acid phosphatase and MMPs, resulting in the breakdown of the bone structure and release of mineral components.^29,30^ The mineralized substances and organic materials generated from bone resorption need to be absorbed and cleared by surrounding cells, including osteoblasts and stromal cells.^31^ Under normal circumstances, bone resorption and bone formation are balanced, maintaining the health and structural stability of the skeletal system^32^ (Fig. 1). During sleep and circadian rhythm disturbances, the balance of bone metabolism may be disrupted. Epidemiological studies indicate that individuals with circadian rhythm dysregulation caused by shift work have an increased risk of low bone mineral density and fractures.^33,34^ Experimental studies of rats and healthy men show that sleep and circadian disruption may impair bone formation.^35,36^ However, the mechanisms by which circadian rhythms influence bone metabolism have not been fully elucidated.

Daily variations in normal bone physiology indicate that bone turnover has a time-dependent component essential for optimal bone health. The skeletal system possesses a circadian rhythm, as evidenced by luciferase reporter experiments indicating that the osteocalcin promoter’s expression in bone tissue exhibits 24-h rhythmic oscillation characteristics.^14,37,38^ Notably, the levels of BMP2 and RUNX2, which are pivotal transcription factors involved in bone formation, undergo changes in response to variations in light cycles and melatonin.^39^ Also, diurnal fluctuations are observed in serum bone turnover m5zarkers.^25^ Synchronous circadian rhythm promotes the formation of healthy cartilage and bone structure.^26,38,40^ Bone resorption markers, including N-terminal cross-linked telopeptide of type I collagen (NTX) and C-terminal cross-linked telopeptide of type I collagen (CTX), also have a clear sinusoidal rhythm across the 24 h day similar to that of bone formation markers.^41,42^

Recent years have witnessed an increasing number of studies elucidating the association between *Bmal1* and bone/cartilage metabolism. *Bmal1* plays a crucial role in embryonic development and intramembranous ossification. Mice with *Bmal1* gene deficiency exhibit the phenotype of low bone mass, characterized by diminished microstructures like bone cortex and trabecular bone, diminished bone density, as well as a reduced count of active osteoblasts and osteocytes within the organism.^43^ The deletion of *Bmal1* in mice results in morphological deformities and premature aging of the mandibular condyle,^44^ and degradation of knee joint cartilage.^45^ Conversely, osteoclast-specific deletion of *Bmal1* in mice exhibits a high bone mass phenotype due to inhibited bone resorption.^40^ In vitro co-culture experiments demonstrate that overexpression of *Bmal1* in osteoblasts upregulates the expression of BMP, RUNX2, and osteocalcin, positively regulating osteogenesis.^46^ Deletion of *Bmal1* in chondrocytes not only impairs chondrocyte survival and secretion function but also increases the expression of cartilage matrix-degrading enzymes.^47^ Moreover, extensive studies have indicated the involvement of *Bmal1* in the regulation of MSCs differentiation. For instance, BMSCs isolated from *Bmal1* gene knockout mice exhibit reduced capacity for osteogenic differentiation in vitro.^43^ The BMAL1-CLOCK complex, conversely, promotes osteogenic differentiation of MSCs by upregulating P300 to acetylate histone 3 in the *Runx2* promoter.^48^

However, conflicting results have also been reported. Fu et al. observed increased osteogenic-related parameters and osteoblast numbers in *Bmal1* knockout mice.^49^ In vitro experiments demonstrated enhanced osteogenic differentiation capacity of BMSCs with inhibited *Bmal1* expression,^50^ suggesting a negative role of *Bmal1* in the regulation of osteogenesis. These inconsistent conclusions may be explained by the aging process; in these sets of animal experiments, mice aged from 2 to 5 months, wherein early inhibition of bone formation might be masked. Additionally, differences in signals within in vivo and in vitro experimental environments could lead to disparate outcomes. Furthermore, research has shown that colon epithelial cell-specific deletion of *Bmal1* also impairs trabecular and cortical bone formation in male mice, evidenced by decrease of serum bone formation marker procollagen type 1 N-terminal propeptide (P1NP) and dynamic histomorphometry,^51^ indicating a complex relationship between *Bmal1* and bone and cartilage metabolism.

## Signaling pathways by which *Bmal1* modulates bone and cartilage metabolism

**Fig. 2:**
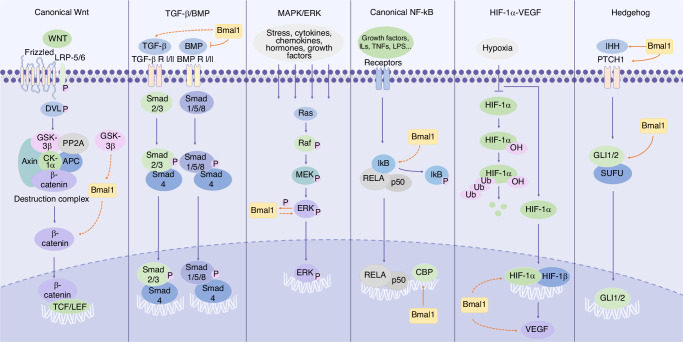
Schematic diagram of *Bmal1* involving in different signaling pathways. We include the key components of these pathways and mark the effect targets of *Bmal1*. Notably, due to the lack of conclusive results regarding the binding sites and specific regulatory mechanisms of *Bmal1*, we use dashed arrows to indicate the indirect effect of *Bmal1*, either up- or down-regulating the expression of downstream molecules, whereas solid arrows/inhibition symbols to represent relatively clear direct promotion/inhibition effects

The metabolism of bone and cartilage is regulated by numerous signaling pathways, such as Wnt, transforming growth factor β (TGF-β)/BMP, mitogen-activated protein kinase (MAPK), and hedgehog (Fig. 2). Current research suggests that these signaling pathways are involved to varying degrees in the regulation of bone and cartilage metabolism by clock genes^52–55^(Table 1). Moreover, there exists crosstalk among these pathways, for instance, the crosstalk between TGF-β and Wnt, MAPK, and hedgehog pathways.^56,57^ Currently it is challenging to attribute the regulation of bone and cartilage metabolism by *Bmal1* to one single pathway, but rather it is accomplished by a synergistic signaling network and numerous regulatory factors working together.

**Table 1:** The various signaling pathways and regulatory mechanisms through which *Bmal1* regulates bone and cartilage metabolism

Signaling pathways	Cells/disease models	Effects	References
Wnt/β-catenin signaling pathway	Chondrocytes	*Bmal1* knockdown activated β-catenin expression, downregulated GSK-3β expression, and stimulated an inflammatory response	^73^
	BMSCs	*Bmal1* suppressed Wnt/β-catenin pathway, negatively regulated the osteogenic differentiation ability of BMSCs	^52,66^
	Diabetic BMSCs	*Bmal1* overexpression activated the Wnt/β-catenin signaling pathway and restored BMSC osteogenic capacity by suppressing GSK-3β to some extent	^70^
	NIH-3T3 cells	*Bmal1* overexpression increased β-catenin expression and enhanced cell proliferation rate	^65^
TGF-β/BMP signaling pathway	MC-3T3 cells	*Bmal1* over expression upregulated BMP2, RUNX2, OC expression, promoted osteoblast differentiation	^46^
	BMSCs	*Bmal1* overexpression upregulated BMP2 expression, promoted osteogenic differentiation in BMSCs	^84^
	Osteoblasts	*Bmal1* deficiency activated the BMP2/SMAD1 signaling pathway, increasing osteoblast activity in the cortical area of adult mice; BMAL1 is a transcriptional silencer of BMP2 by directly binding to the *Bmp2* promoter	^53^
	Human articular chondrocytes	*Bmal1* deficiency reduced the levels of phosphorylated SMAD2/3 but increased p-SMAD1/5 activity	^26^
	Ligament-derived and embryonic fibroblasts	*Bmal1* knockdown upregulated osteogenic markers as well as TGF-β/BMP pathway signals, inducing endochondral ossification in heterotopic ossification of tendons and ligaments	^85^
MAPK/ERK signaling pathway	Chronic sleep deprivation rats	Chronic sleep deprivation activated p-ERK expression, resulting in upregulation of MMP-1, -3, and -13, and temporomandibular joint damage	^101^
	Mandibular condylar chondrocytes	*Bmal1* inhibition activated ERK phosphorylation, while ERK inhibition did not affect *Bmal1* expression; *Bmal1* overexpression reversed temporomandibular joint osteoarthritis in rats	^54^
NF-κB signaling pathway	Diabetic BMSCs	*Bmal1* may rescue diabetic BMSC osteogenic function by inhibiting the NF-κB pathway to some extent	^114^
	Chondrocytes	IL-1β reduced *Bmal1* expression through NF-κB activation	^115^
Hedgehog signaling pathway	Chondrocytes	BMAL1 directly bound to *Ptch1* and *Ihh* promoter region to activate hedgehog pathway, regulating chondrogenesis and endochondral ossification of mandibular condylar cartilages	^55^
HIF-1α-VEGF signaling pathway	Chondrocytes	*Bmal1* deficiency upregulated the expression of HIF1α, VEGF, MMP13 and RUNX2, leading to decrease of chondrocyte proliferation	^140^
TTK/MDM2/ H2Bub1 axis	MSCs	*Bmal1* targeted the circadian-controlled gene TTK to phosphorylate MDM2 (murine double minute 2) and modulate H2Bub1 (one histone) levels to positively affect the osteogenic capacity of MSCs	^186^

### Wnt signaling pathway

The Wnt signaling pathway participates in the regulation of various biological processes.^58,59^ Among these, the canonical Wnt pathway, wherein Wnt signaling regulates downstream pathways through β-catenin as a key node, primarily governs cell proliferation and influences cell fate during development.^60^ The pathway is activated on binding of WNT ligands to Frizzled (FZD) receptors. Low-density lipoprotein receptor-related protein-5/6 (LRP-5/6) is phosphorylated, and the dishevelled (DVL) in the cytoplasm is recruited to the membrane and phosphorylated, too. Once DVL is phosphorylated, β-catenin is released from the destruction complex which sequesters and causes β-catenin degradation, and β-catenin can now translocate to the nucleus and enable the transcription of target genes along with T cell factor (TCF)-lymphoid enhancer factor (LEF) transcription factors.^59,61^ The canonical Wnt signaling pathway plays a crucial role in regulating the proliferation and differentiation of BMSCs and osteoprogenitor cells, as well as the bone resorption function of osteoclasts, exerting significant effects on bone development and maintenance.^62^ Many regulatory factors are involved in this process. For example, sclerostin is a crucial osteocyte-derived Wnt-β-catenin signaling antagonist, which can bind to the LRP-5/6 receptor and subsequently inhibit the Wnt–β-catenin pathway involved in bone metabolism.^63^ Loss of sclerostin promotes osteoarthritis in mice through Wnt-β-catenin signaling pathway.^64^ These key pathway regulators may become important targets for future research.

Previous studies have indicated the process-specific involvement of the clock gene *Bmal1* in the activation of the Wnt/β-catenin pathway by influencing β-catenin (Fig. 2). Overexpression of *Bmal1* in NIH-3T3 cells increased β-catenin expression and significantly enhanced cell proliferation rate, suggesting that *Bmal1* may promote cell proliferation by activating the canonical Wnt pathway.^65^ Another study showed that transfection of cells with Wnt signaling inhibitor Dkk1 resulted in a significant decrease in mRNA and protein levels of *Bmal1* after 7 days of osteoinduction, indicating the process-specific involvement of *Bmal1* in osteoinduction. Furthermore, *Bmal1* overexpression partially rescued the decreased osteogenic capacity induced by Dkk1, with a slight increase in Wnt/β-catenin signaling pathway activity, suggesting that *Bmal1* may positively regulate osteogenesis through the Wnt/β-catenin pathway, although the specific mechanism and interaction between *Bmal1* and the Wnt/β-catenin pathway remain unclear.^66^

Glycogen synthase kinase 3β (GSK-3β), a serine/threonine protein kinase, is a critical negative regulator of the Wnt/β-catenin pathway that targets β-catenin for ubiquitin-mediated degradation, ultimately leading to the inactivation of the Wnt/β-catenin pathway.^67^ Studies have shown that the expression pattern of GSK-3β was similar to that of Rev-erbα, the *Bmal1* expression inhibitor. Overexpression of Rev-erbα inhibited the activity of the Wnt/β-catenin pathway, while the expression trend of GSK-3β was similar to that of Rev-erbα.^68^ In GSK-3β knockdown mouse embryonic fibroblast cells, upregulation of *Bmal1* expression was observed.^69^ Li et al. studied the relationship between GSK-3β, *Bmal1*, and the Wnt/β-catenin pathway in a model of type 2 diabetes-induced inhibition of BMSC osteogenesis. They found that GSK-3β restrained the translocation of β-catenin into the nucleus and downregulated the expression of TCF3 therein, resulting in diminished formation of the β-catenin/LEF/TCF complex. Consequently, this suppression of the Wnt/β-catenin pathway inhibited BMSC proliferation and osteogenic differentiation. Meanwhile, *Bmal1* overexpression can inhibit this negative regulatory factor to some extent and activate the Wnt/β-catenin signaling pathway and restore BMSC osteogenic capacity.^70^ But there is no definitive consensus on the regulatory relationship between *Bmal1* and GSK-3β,^71,72^ yet it was insufficient to conclude that the activation of the Wnt/β-catenin pathway is caused by the interaction between *Bmal1* and GSK-3β.

However, some studies have reached the opposite conclusion that decrease of *Bmal1* can activate Wnt/β-catenin pathway. In a study of rat knee cartilage degeneration, chronic circadian rhythm disruption and osteoarthritis environment significantly reduced the protein expression of BMAL1 in chondrocytes but markedly activated the Wnt/β-catenin signaling pathway. Silencing *Bmal1* in vitro in chondrocytes upregulated the protein and mRNA expression of β-catenin, downregulated the protein and mRNA expression of GSK-3β, and induced inflammatory response in cells.^73^ RORs have been shown to promote *Bmal1* transcription^74^ and are also one of the downstream regulatory factors of the Wnt/β-catenin pathway,^75^ indicating that nuclear transcription factors may be involved in *Bmal1*’s influence on the Wnt signaling pathway. Studies have also supported that *Bmal1* may suppress the Wnt/β-catenin signaling pathway by downregulating Rorα expression or upregulating Rev-erbα expression, affecting BMSC proliferation and osteogenic differentiation, thereby regulating BMSC aging.^52^

These results suggest that the interaction mechanism between *Bmal1* and the Wnt/β-catenin pathway may vary in different microenvironments and may be tissue or cell-specific. In different environments stimulated by inflammation or other cytokines, the expression of clock genes may vary.

### TGF-β/BMP signaling pathway

The TGF-β superfamily includes TGF-βs, activins, BMPs, and other related proteins, with TGF-β/BMP signaling being crucial for inducing bone formation throughout the organism. TGF-βs and BMPs transduce signals through SMAD-dependent and -independent pathways.^76^ Through the SMAD-dependent pathway, TGF-β or BMP binds to type I and II receptors on the cell surface, leading to phosphorylation of SMAD proteins (critical intracellular signaling molecules in the TGF-β signaling pathway). Phosphorylated SMADs then form complexes with SMAD4, translocate to the nucleus, and trigger the transcription of *Runx2* to regulate osteogenesis.^56,77^ The TGF-β pathway can interact with the Wnt pathway; on one hand, TGF-β can upregulate the expression of Wnt molecules (Wnt-2, -4, -5a, -7a, -10a) and Wnt co-receptor LRP5, inhibits the expression of β-catenin inhibitor Axin1/2, and promotes Wnt/β-catenin signaling pathway transduction.^78^ TGF-β can also induce Wnt/β-catenin signaling pathway expression through TGF-β/SMAD3 axis.^79^ On the other hand, Wnt signaling induces RUNX2-mediated expression of TGF-β type I receptor in a β-catenin-independent manner, promoting TGF-β signaling,^80^ thereby synergistically stimulating osteoblast and chondrocyte differentiation.

Clock genes have been shown to be closely associated with TGF-β/BMP, and *Bmal1* can activate the TGF-β/BMP pathway. For example, *Bmal1* controls TGF-β and BMP signaling during brown adipose tissue generation.^81^ TGF-β is controlled by BMAL1/CLOCK heterodimers, and its circadian rhythm is lost in skeletal muscle tissue with *Bmal1* deficiency.^82^ Also, disruption of *Bmal1* in mice promotes cancer metastasis by TGF-β-dependent pathway.^83^ In MC-3T3 cells and BMSCs overexpressing *Bmal1*, it was found that *Bmal1* upregulates the transcription of *Bmp2*, thereby upregulating RUNX2 and BMP2 expression, promoting osteogenic differentiation.^46,84^

However, the regulatory effects of *Bmal1* on *Bmp* genes are inconsistent, too. Research showed that disruption of *Bmal1* led to activation of the BMP signaling pathway in chondrocytes and adipocytes, whereas osteoblasts, chondrocytes, and adipocytes all originate from the same progenitor cells.^26,81^ In osteoblasts, Qian et al. found that *Bmal1* deficiency increased BMP2 expression levels and promoted SMAD1 phosphorylation, activating the BMP2/SMAD1 signaling pathway, increasing osteoblast activity in the cortical area of early adult mice, leading to relatively unaffected high trabecular bone mass. With increasing age, higher trabecular bone mass persists, while cortical bone mass decreases, accompanied by spinal deformities and intervertebral disc deformities in mice. Furthermore, Qian et al. revealed that BMAL1 is a transcriptional silencer of BMP2 by directly binding to the *Bmp2* promoter (Fig. 2).^53^

Clock gene efficacy varies in different age models as well. For example, *Bmal1*-deficient mice exhibit disrupted circadian rhythms and premature aging. In this aging model, *Bmal1* deficiency significantly upregulates the mRNA expression levels of osteogenic markers *Opn*, *Alp*, *Ocn*, *Col1a1*, and *Runx2*, as well as genes related to the TGF-β/BMP pathway (including *Bmp1*, *Bmp2*, *Smad1*, *Smad5*, *Smad2*, and *Smad3*), promoting chondrogenic and osteogenic differentiation of embryonic fibroblasts (a type of multipotent progenitor cell capable of differentiating into the osteogenic lineage) in mouse ligaments and tendons with age, leading to enhanced progressive heterotopic ossification.^85^ Currently, there is limited research on aging models, and further investigation is needed to elucidate the specific mechanisms of clock genes in different age models.

### MAPK/ERK signaling pathway

The MAPK/extracellular signal-regulated kinase (ERK) signaling pathway targets over 600 intracellular substrates, enabling rapid responses to injury stimuli and coordinating fundamental cellular activities, including cell survival, migration, proliferation, growth, transcription, cycle arrest and death.^86–88^ The MAPK pathway is interconnected with the TGF-β pathway.^89,90^ Through SMAD-independent pathways, TGF-β and BMP activate MAPK signaling and positively regulate the expression and function of RUNX2, promoting MSC differentiation.^91^ Phosphorylation of ERK is a hallmark of MAPK activation.^92^ A considerable amount of research has demonstrated that the MAPK signaling pathway simultaneously serves as input and output pathways in the circadian clock.^93–95^ ERK can directly interact with components of the circadian oscillator and phosphorylate them, including BMAL1, CLOCK, CRY1, and CRY2, playing a significant role in maintaining circadian rhythms.^96,97^

Studies have shown a direct correlation between *Bmal1* and p-ERK expression. ERK is a key factor in inducing many MMP subtypes (e.g., MMP-1, -3, -13), and the upregulation of MMPs is closely related to temporomandibular joint disorders, which leads to loss of collagen and glycosaminoglycans.^98–100^ In a rat model of sleep deprivation, the MAPK/ERK pathway was activated, leading to enhanced phosphorylation of ERK, upregulation of downstream MMP-1, MMP-3, and MMP-13 mRNA and protein levels, finally resulting in temporomandibular joint damage. This damage can be rescued through applying selective ERK inhibitor.^101^Moreover, under conditions of circadian rhythm disruption, dysregulation of *Bmal1* gene expression in chondrocytes was observed alongside enhanced ERK phosphorylation and upregulated expression of the inflammatory factor IL-6. However, with the overexpression of *Bmal1*, p-ERK was inhibited. Conversely, inhibiting ERK expression led to decreased expression of p-ERK and downstream MMP3/13, while *Bmal1* expression remained unaffected. These results suggest that dysregulation of *Bmal1* expression may affect the expression of MMPs and IL-6 through activation of the MAPK/ERK pathway, leading to cartilage tissue damage.^54^ In bones and cartilage tissues other than the temporomandibular joint, the interaction between the MAPK/ERK pathway and *Bmal1* awaits further investigation.

### NF-κB signaling pathway

The NF-κB transcription factor plays a critical role in immunity, inflammation, cell proliferation, differentiation, and survival.^102–104^ The NF-κB family consists of five related transcription factors, including RELA (p65), RELB, c-Rel、p105/p50 and p100/p52.^105,106^ The RELA/p50 complex pathway is canonical. Under steady states, RELA/p50 complexes are sequestered in the cytoplasm by IκB proteins (the inhibitor of NF-κB).^107^ Under inflammatory stimulation, cytoplasmic RELA/p50 complexes translocate to the nucleus and induce rapid responses through target gene transcription.^108^ The hallmark of activation of the canonical NF-κB pathway is the phosphorylation of IκB.^109^ RELB and p52 form heterodimers in the non-canonical NF-κB pathway.^104^

NF-κB has been shown to regulate cellular circadian rhythms, and *Bmal1* can, in turn, regulate NF-κB signaling pathway through IκB and CBP (CREB-binding protein).^110,111^ In a human U2OS cell model, changes in the NF-κB subunit RELA altered the expression of core clock genes, shortening cell cycle length and inhibiting rhythm amplitudes. Furthermore, NF-κB, similar to the clock repressor CRY1, can directly bind to the transactivation domain of BMAL1, forming a complex with BMAL1/CLOCK and indirectly inhibiting CCG E-box element transcription.^112^ On the other hand, it has been demonstrated that *Bmal1* can activate the NF-κB signaling pathway by increasing the phosphorylation of IκB. Also, overexpression of *Bmal1* recruited CBP to enhance p65 activity which further activated the NF-κB signaling pathway, thus regulating the expression of its downstream target genes and promoting the invasion and metastasis of breast cancer cells.^113^

Research has attempted to explore the regulation of bone/cartilage metabolism by *Bmal1* and the NF-κB signaling pathway in different microenvironments. It was observed that under diabetic conditions, BMSCs exhibited osteogenic inhibition, decreased *Bmal1* expression, and loss of inhibitory factor κB expression, indicating activation of the NF-κB signaling pathway. Conversely, overexpression of *Bmal1* in BMSCs led to inhibition of the NF-κB signaling pathway. These results suggest that under diabetic microenvironments, *Bmal1* may rescue BMSC osteogenic function by inhibiting the NF-κB pathway to some extent.^114^

Additionally, research has investigated the relationship between the NF-κB signaling pathway and clock genes in an inflammatory microenvironment. The results showed that IL-1β treatment reduced *Bmal1* expression in chondrocytes, while phosphorylated p65 levels increased, indicating increased NF-κB activation. Inhibiting the NF-κB pathway prevented the decrease in *Bmal1* expression induced by inflammation.^115^ However, the specific regulatory mechanisms of *Bmal1* and the NF-κB signaling pathway in cell bone and cartilage metabolism under different microenvironments require further investigation.

### Hedgehog signaling pathway

The hedgehog pathway plays a crucial role in bone formation, repair, and homeostasis.^116–118^ It regulates MSC osteogenic differentiation either individually or in conjunction with signals such as Wnt, BMP, and parathyroid hormone-related proteins, in bone formation and repair processes.^119–121^ Dysregulation of the hedgehog signaling pathway has been implicated in bone-related diseases such as osteoarthritis, osteoporosis, and fractures.^122–125^ The main components of this pathway include the hedgehog ligands (with Indian hedgehog, IHH, as the core ligand), the Patched (PTCH) receptor, and the transcription factor GLI. Its activation relies on the binding of hedgehog protein to its receptor PTCH.^126^

Multiple studies have shown that BMAL1 can regulate hedgehog signal transduction and influence downstream cascades by directly binding to the promoters of PTCH1, IHH, or GLI2.^55,127^ It has been observed that in chondrocytes with specific *Bmal1* deficiency, the expression of *Ihh*, *Ptch1*, and *Gli1* is significantly downregulated, leading to decreased chondrocyte proliferation and reduced cartilage matrix formation, increased chondrocyte apoptosis, delayed and reduced cartilage formation and endochondral ossification in the condylar process of the mandible in mice, which can be partially rescued by overexpressing *Ptch1* during prepubescence or prepubertal period. These results indicate that the hedgehog pathway is tightly controlled by *Bmal1* during the development of the mandibular condylar process.^55^

### HIF-1α-VEGF signaling pathway

Cartilage tissue is a unique avascular and hypoxic tissue in the body, requiring various factors to regulate and maintain its normal developmental and metabolic processes, such as the hypoxia-inducible factors α (HIFα) family composed of HIF-1α, HIF-2α, and HIF-3α.^128–130^ Specific loss of HIF-1α in cartilage induces significant cell apoptosis in the central region.^131^ BMAL1, as a participant of the HIF protein family’s β subunit, interacts with the α subunit, regulating cells to adapt to different transcriptional and protein level environments and sharing major protein structural domains with HIF-1α.^132–134^

Studies have shown that *Bmal1* can interact with HIF-1α or HIF-2α, mediating various key physiological functions. For example, in mouse nucleus pulposus cells, *Bmal1* controls the transcriptional activity of HIF-1α and influences HIF-1α target genes expression.^135^ In addition to independently regulating cellular responses to hypoxia, HIF-1α and HIF-2α also bind to hypoxia response elements to initiate downstream factors such as VEGF transcriptional activity.^136,137^ VEGF is a classical target of HIF and plays a central role in cartilage development, particularly in the transition from avascular cartilage growth plates to highly vascularized bone (known as osteogenesis-angiogenesis coupling).^138,139^ In the study by Ma et al., mice with cartilage-specific *Bmal1* knockout exhibited delayed development of growth plate cartilage during puberty, decreased chondrocyte proliferation, and activation of cell apoptosis. Meanwhile, *Bmal1* deletion attenuated the expression of VEGF and HIF-1α in growth plate cartilage cells and enhanced the levels of MMP13 and RUNX2. These findings suggest that *Bmal1* may play a role in cartilage development by regulating the HIF-1α-VEGF signaling pathway.^140^

In conclusion, further studies are needed to clarify: (1) The specific regulatory mechanisms between *Bmal1* and various signaling pathways. (2) The mechanisms underlying the inconsistent conclusions across different research models. (3) The expression of circadian rhythm genes in aging models and the mechanisms by which they regulate bone/cartilage metabolism. (4) The network of synergistic regulation by *Bmal1* and other circadian rhythm genes in modulating multiple bone metabolism pathways.

## Control of *Bmal1* regarding bone/cartilage metabolism

Known *Bmal1* controls regarding bone/cartilage metabolism include: micro RNAs (miRNAs), drugs including melatonin and fluoride, some molecules including sirtuin 1 (SIRT1) and lysine-specific demethylase 6B (KDM6B), and some hormones, such as parathyroid hormone (PTH) and norepinephrine (NE). Most of them exert their effects by directly binding to the *Bmal1* promoter. New attempts have also been made in the field of tissue engineering by developing novel biomaterials to control *Bmal1* expression and facilitate bone remodeling. A brief overview of these regulatory factors is as follows. Notably, here we mainly focus on peripheral controls. Control of *Bmal1* by central oscillator can be reviewed in other excellent papers.^141,142^

### MiRNAs

MiRNAs are vital for posttranscriptional regulation in the peripheral clock network. They can be chemically modified and easily delivered to the target site, making them promising candidates for therapeutic applications.^143^ Through dual-luciferase reporter assay or chromatin immunoprecipitation assay, now several miRNAs are found able to directly target *Bmal1*. For example, in an intervertebral disc disease (IDD) model, a novel miRNA *hsa-let-7f-1-3p* was found to directly bind to *Bmal1* 3’UTR and can reduce BMAL1 expression, induce autophagy in nucleus pulposus cells, ultimately mediating disc degeneration.^144^ *miR-155-5p*, also directly targeting *Bmal1* 3’UTR, was reported to repress *Bmal1* expression and inhibit the proliferation and osteogenic differentiation of BMSCs, along with the expression and nuclear translocation of YAP and TAZ.^145^ *miR-142-3p*, an age-dependent miRNA, exhibited similar regulatory function to *miR-155-5p*, which can affect the expression of BMAL1/YAP and reduce the osteogenic potential of BMSCs.^146,147^ Several other miRNAs also have shown potential in regulating *Bmal1* expression, such as *miR-211*, which targeted the proximal promoter/5’UTR region of *Bmal1* and regulated it via RNA-induced transcriptional silencing^148^; *miR-223*, targeting *Bmal1* 3’UTR, inhibited *Bmal1* expression while activated MMP-1, -9 expression in macrophages by *Mycobacterium tuberculosis* infection^149^; and *miR-27b-3p*, which can also suppress *Bmal1* by binding to the 3’UTR.^150^ They may become the potential regulatory miRNAs of bone/cartilage circadian clock.

### Drugs

Melatonin and melatonin receptors (MTRs) play an important role in mediating circadian rhythm.^151^ Melatonin has long been recognized for its regulation of circadian rhythms, and exogenous melatonin is not toxic to animals over a wide dose range.^152^ Melatonin is able to maintain the rhythmic homeostasis of the bone microenvironment.^153^ It is reported that melatonin modulates the rhythmic expression of *Bmal1*,^154^ and can upregulate *Bmal1* expression in RAW264.7 cells, induce apoptosis, suppress cell oxidative damage, and consequently improved postmenopausal osteoporosis in mice.^155^ MTR1 was found vital for the circadian rhythm in the chondrification center. MTR1 in chondrocytes phosphorylated AMPKβ1 to transmit rhythm signals, the phosphorylated AMPKβ1 can further phosphorylate and destabilize CRY1, thus upregulating the expression of *Bmal1*. Consequently, the osteogenesis abnormity caused by circadian dysrhythmia can be partially rescued.^156^

Fluoride is an essential trace element for the human body that is widely found in nature. Excessive fluoride intake can lead to chronic systemic diseases characterized by skeletal fluorosis and dental fluorosis. Studies show that fluoride is involved in the proliferation and differentiation of chondrocytes.^157,158^ In chondrocytes treated with NaF, the circadian rhythm was disrupted, along with downregulation of *Bmal1* expression, inhibition of chondrocyte viability and delay of chondrocyte differentiation. While overexpression of *Bmal1* partially reversed this process, suggesting fluoride a negative effector during chondrogenesis.^159^

### Molecules

SIRT1 is a conserved nicotinamide adenine dinucleotide(NAD)^+^ -dependent protein deacetylase that participates in many physiological pathways by deacetylating target proteins.^160^ SIRT1 can modulate the circadian rhythm by controlling the acetylation levels of BMAL1.^161^ Another study showed that SIRT1 can bind to the *RORα*-binding sites (RORE) with PPARγ coactivator-1α (PGC-1α) at the proximal *Bmal1* promoter region to positively regulate *Bmal1* expression in the SCN, and SIRT1-deficient mice exhibited senescence.^162^

KDM6B is reported to promote osteogenic differentiation of human MSCs through removal of trimethylated histone 3 lysine 27 and activation of the transcription of BMP2.^163^ KDM6B can directly target the promoter region of *Bmal1* in bone marrow-derived macrophages (BMDMs) and activate *Bmal1* transcription, which further reduced macrophage pyroptosis and the M1/M2 ratio through inhibiting TLR2/NF-κB signaling pathway, ultimately promoting osteogenic differentiation of BMSCs.^164^

Stress stimuli can lead to alterations in circadian rhythm expression as well. Studies have showed that orthodontic force, which stimulates periodontal ligament cells (PDLCs) to release biomechanical signals to initiate alveolar bone remodeling, upregulates *Bmal1* expression in PDLCs in manners dependent on ERK and activator protein 1 (AP1). AP1 can directly interact with the *Bmal1* promoter and activate C-C motif chemokine 2 and RANKL transcription in PDLCs, which consequently recruits monocytes to differentiate into osteoclasts, promotes bone remodeling and orthodontic tooth movement.^165^ In another IDD model induced by abnormal mechanical loading, the intrinsic circadian clock in the intervertebral disc was dampened, and the *Bmal1* expression could be partially rescued by RhoA/ROCK pathway (a crucial pathway in mechanotransduction^166^) inhibition Y-27632 and melatonin.^167^

Some hormones are also proved to be involved in the regulation of clock genes. PTH stimulates osteoblast differentiation and proliferation,^168^ while NE suppresses bone formation via osteoblast β-adrenergic receptor (βAR) signaling.^169^ *Bmal1* is involved in both processes and serves as their common target. Specifically, NE/βAR signaling activation inhibited the upregulation of *Bmal1* expression induced by intermittent PTH in osteoblasts, thus impairing PTH-induced bone anabolism.^170^

### Biomaterials

In the field of tissue engineering, researchers have also paid their attention to circadian rhythm regulation. In a study on bone defect regeneration, reactive oxygen species (ROS) scavenging and responsive prolonged oxygen-generating hydrogels (CPP-L/GelMA), a bone microenvironment regulative hydrogel, was developed to modulate hypoxic conditions in bone defects region. CPP-L/GelMA was able to rescue the decreased expression of *Bmal1* and activate the autophagy of osteoblasts, thus facilitating bone regeneration in vivo.^171^

We summarize the above regulatory factors that control *Bmal1* in Table 2 for quick review. Overall, there is still a lack of research on the potential regulatory factors of clock genes as well as their regulation mechanism. How to identify promising regulators and apply them to treat bone/cartilage diseases or defects are important directions for future research.

**Table 2:** Upstream control of *Bmal1*

Category	Regulatory factors	Binding sites	Disease models and regulatory effects	References
miRNAs	*has-let-7f-1-3p*	Directly bind to *Bmal1* 3’UTR	Downregulate *Bmal1* protein expression, induce IDD	^144^
	*miR-155-5p*	Directly bind to *Bmal1* 3’UTR	Inhibit *Bmal1* expression and BMSCs proliferation and osteogenic differentiation, promote cell apoptosis and senescence	^145^
	*miR-142-3p*	Directly bind to *Bmal1* 3’UTR	Inhibit *Bmal1* expression, decrease the osteogenic potential of BMSCs	^146^
	*miR-211*	Directly bind to *Bmal1* 5’UTR	Transiently suppress *Bmal1* expression	^148^
	*miR-223*	Directly bind to *Bmal1* 3’UTR	Inhibit *Bmal1* expression, activate MMPs expression in macrophages by *M. tuberculosis* infection	^149^
	*miR-27b-3p*	Directly bind to *Bmal1* 3’UTR	Inhibit *Bmal1* expression	^150^
Molecules	SIRT1	Deacetylate BMAL1 at Lys537	Modulate circadian rhythm	^161^
		Sirt1/PGC-1α complex directly target RORE	Upregulate *Bmal1* in SCN, associated with aging	^162^
	KDM6B	Directly bind to *Bmal1* E-box	Increase *Bmal1* expression in BMDMs, induce osteogenic differentiation of BMSCs	^164^
	AP1	Directly interact with *Bmal1* promoter	Increase *Bmal1* expression in PDLCs, promote alveolar bone remodeling	^165^
	PTH	/	Increase *Bmal1* expression in the osteoblasts from osteoporotic fracture mice	^170^
	NE	/	Activate βAR signaling, inhibit *Bmal1* expression, suppress fracture healing	^170^
Drugs	Y-27632	/	Indirectly reverse *Bmal1* expression in IDD by inhibiting RhoA/ROCK pathway	^167^
	AMPKβ1 agonist	Indirect effect through phosphorylation of CRY1	Increase *Bmal1* expression, alleviate the abnormity of endochondral ossification caused by circadian dysrhythmias	^156^
	Melatonin	/	Indirectly reverse *Bmal1* expression in IDD by inhibiting RhoA/ROCK pathway	^167^
		/	Induce macrophage apoptosis via the BMAL1/ROS/MAPK-p38 pathway, improve postmenopausal osteoporosis	^155^
		/	Upregulate *Bmal1* in mouse primary chondrocytes	^154^
	Fluoride	/	Suppress *Bmal1* expression, inhibit chondrocyte viability, delay chondrocyte differentiation	^159^
Biomaterials	CPP-L/GelMA hydrogel	/	Improve *Bmal1* expression in osteoblasts, promote osteogenic differentiation by enhancing autophagy, and facilitate bone regeneration	^171^

## Mouse models for studying the role of *Bmal1* in bone/cartilage diseases

*Bmal1*-deficient mice exhibit different extents of manifestations that are related to bone/cartilage metabolism, and global *Bmal1* knockout mice has been employed to depict the specific role of *Bmal1* in regulating bone/cartilage diseases. In recent years, several conditional *Bmal1*-deficient mouse models have been developed, and they helped to illustrate the exact function of Bmal1 in regulating diverse types of cells; such mouse models include the following: *Bmal1*^*-/-*^ mice, *Col1a1-Cre*^*ERT2*^*:Bmal1*^*flox/flox*^ mice, *Bmal1*_*osx*_^*-/-*^ mice, *Bmal1*^*f/f*^*.Prx1-cre*, *Col6a1-Bmal1*^*-/-*^, and *Col2a1-CreER*^*TM*^*:Bmal1*^*flox/flox*^. Notably, *Bmal1* has also been conditionally deleted in other organs to investigate the crosstalk between peripheral clock signaling and bone/cartilage metabolism; such mouse models include the following: *Ts4-Cre;Bmal1*^*fl/f*^, *Bmal1*_Int_^*-/-*^, and iMS*Bmal1*^*-/-*^. Here, we summarize the knockout strategies of these *Bmal1*-related mouse models and exemplify how these genotypes imitate typical manifestations of bone/cartilage diseases, to help study the etiological and pathological role of *Bmal1* (Table 3).

**Table 3:** Overview of *Bmal1*-related mouse models

Cell	Genotype	Phenotype	References
Full knock out	*Bmal1*^*-/-*^	Facial dysmorphism; skeletal mandibular hypoplasia; decreased bone mineral density in interalveolar septum and mandibular inferior cortex	^55,172,173^
Osteoblast-specific	*Col1a1-CreERT2:Bmal1*^*flox/flox*^	Increased the number of osteoblasts and expression of bone formation markers; elevated trabecular osteoblast activity	^53^
	*Bmal1*_*osx*_^*-/-*^	Enhanced osteoblast-dependent regulation of osteoclastogenesis; decreased bone mass	^174^
Osteoclast-specific	*Bmal1*^*osc-/-*^	Declined osteoclast differentiation; increased bone mass	^40^
		No significant difference	^25^
Mesenchymal Cells-Specific	*Bmal1*^*f/f*^*.Prx1-cre*	Low trabecular bone mass; increased bone turnover	^45^
	*Col6a1-Bmal1*^*-/-*^	Thickening of the synovial subintimal; nodular chondroid metaplasia; spur formation at the condyles of the distal tibia	^177^
Chondrocyte-Specific	*Col2a1-Bmal1*^*-/-*^ or *Col2a1-CreER*^*TM*^*:Bmal1*^*flox/flox*^	Decreased osteogenic differentiation; disorganization of extracellular matrix structure; cartilage degradation and chondrocyte apoptosis	^26,178^
Skeletal Muscle- Specific	iMS*Bmal1*^*-/-*^	Declined joint collagen; elevated bone calcification	^82^
Colon Epithelial Cell-Specific	*Ts4-Cre;Bmal1*^*fl/fl*^	Decreased bone formation	^51^
Intestinal Tissue-Specific	*Bmal1*_Int_^*-/-*^	Increased bone resorption	^179^

### *Bmal1*^*-/-*^

Global deletion of *Bmal1* in mice has been implemented frequently in exploring bone/cartilage diseases, and loss of *Bmal1* has been proved to be closely related to many aberrant manifestations in bone/cartilage. *Bmal1*^*-/-*^ mice demonstrated reduced chondrogenesis and endochondral ossification in mandibular condyle, which may lead to facial dysmorphism.^55^ BMAL1 deficiency also increased osteoclast differentiation and resulted in skeletal mandibular hypoplasia (SMH).^172^ Moreover, bone mineral density in interalveolar septum between the first and second molars and mandibular inferior cortex is significantly decreased in *Bmal1*^*-/-*^ mice.^173^

### Osteoblast-Specific *Bmal1* Knockout Mice

There’s controversy about the role of BMAL1 in regulating the osteoblast activity, and different types of osteoblast-specific *Bmal1* knockout mice suggested inconsistent manifestations. To specifically delete *Bmal1* in osteoblasts, the *Col1a1-Cre*^*ERT2*^*:Bmal1*^*flox/flox*^ conditional knockout mice were obtained by Qian et al. through crossing *Bmal1*^*flox/flox*^ mice with *Col1a1-Cre*^*ERT2*^ transgenic mice, which was based on tamoxifen-inducible *Col1a1* promoter. The result demonstrated that knockout of *Bmal1* in osteoblasts increased the number of osteoblasts and promoted the expression of bone formation markers, leading to elevated trabecular osteoblast activity, which are related to kyphoscoliosis and malformed intervertebral disk in aged mice.^53^ In another research, Takarada et al. generated osteoblast-specific *Bmal1* knockout mice (*Bmal1*_*osx*_^*-/-*^ mice) by crossing *Bmal1*^*flox/flox*^ mice with *Osx–Cre* transgenic mice. Whereas, these mice demonstrated enhanced osteoblast-dependent regulation of osteoclastogenesis, resulting in decreased bone mass reminiscent of global *Bmal1* deletion phenotype.^174^

### Osteoclast-Specific *Bmal1* Knockout Mice

The *Bmal1*^*osc-/-*^ mouse strain, developed by Xu et al. is deficient in BMAL1 expression specifically in osteoclasts. These conditional knockout mice were generated by crossing the *Bmal1*^*flox/flox*^ mice with the transgenic mice under the control of the cathepsin K promoter (*Ctsk-Cre mice*) and nestin promoter (*Nes-Cre mice*). *Bmal1* deletion in osteoclasts leads to decreased expression of osteoclasts makers, such as tartrate resistant acid phosphatase 5 (*Acp5)*, and nuclear factor of activated T cells, cytoplasmic, calcineurin-dependent 1 (*Nfatc1). Bmal1*^*osc-/-*^ mice were not detected altered expression of other circadian genes, revealing that *Bmal1* modulates osteoclastic activity independent of its effect on the oscillating expression of circadian genes. Consequently, *Bmal1*^*osc-/-*^ mice showed declined osteoclast differentiation and a high bone mass phenotype.^40^ Tsang et al. also employed *Ctsk-Cre* driver strain and *Bmal1*^*flox/flox*^ mice to generate osteoclast-specific *Bmal1* knockout mice. Whereas, they found that micro-CT bone parameters of these *Bmal1*^*osc-/-*^ mice were not significantly different from that of *Bmal1*^*flox/flox*^ mice.^25^ Notably, *Ctsk-cre* is also expressed in chondroprogenitor cells and osteoblast progenitors,^175,176^ which may influence the experimental results.

### Mesenchymal cells -Specific *Bmal1* knockout mice

To investigate the role of BMAL1 in regulating fate of mesenchymal cells, Tsang et al. also generated the mesenchymal cells-specific *Bmal1* knockout mice, named *Bmal1*^*f/f*^*.Prx1-cre* through the Cre/loxP site-specific recombination system, which crossed the *Bmal1*^*flox/flox*^ mice with *Prx1-Cre* mice. Notably, *Bmal1*^*f/f*^*.Prx1-cre* mice demonstrated a low trabecular bone mass phenotype due to the increased bone turnover.^25^ Another research deleted *Bmal1* in the joint mesenchymal cells by employing *Col6a1*^*cre/+*^ mice and *Bmal1*^*flox/flox*^ mice. Based on the expression of collagen VI in mesenchymal cells in the ankle joints, *Col6a1-Cre* recombinase is capable of targeting the *Bmal1* in fibroblast-like cells and articular chondrocytes. *Col6a1-Bmal1*^*-/-*^ mice exhibited disruption in ankle joint architecture, which is characterized as thickening of the synovial subintima, nodular chondroid metaplasia, and spur formation at the condyles of the distal tibia. Moreover, deletion of *Bmal1* in mesenchymal cells aggravated the inflammatory response in joint and contributed to inflammatory arthritis.^45^ These findings revealed a crucial role of *Bmal1* in regulating bone/cartilage homeostasis through modulating mesenchymal cells.

### Chondrocyte-Specific *Bmal1* knockout mice

To achieve chondrocyte-specific *Bmal1* deletion, transgenic mouse expressing the *Cre* recombinase under the control of the mouse type II collagen gene (*Col2a1*) was employed by Dudek et al. The *Cre*-mediated recombination was next observed in *Col2a1* expressing cells, especially in chondrocytes. Subsequently, *Col2a1-Bmal1*^*-/-*^ mice were generated via the Cre/loxP site-specific recombination system. Age-related intervertebral disc (IVD) degeneration was observed in *Col2a1-Bmal1*^*-/-*^ mice, characterized by decreased osteogenic differentiation and disorganization of extracellular matrix structure in IVD.^177^ Furthermore, *Col2a1-Bmal1*^*-/-*^ mice suggested progressive degeneration of articular cartilage, revealing the crucial role of BMAL1 in maintaining the cartilage homeostasis and its disruption is implicated with joint diseases such as OA.^26^ Another study generated cartilage-specific *Bmal1* knockout mouse line by breeding *Bmal1*^*flox/flox*^ mice with tamoxifen-inducible *Col2a1-Cre*^*ERT*^ transgenic mice, which expressed *Cre* recombinase directed to chondrocytes. Beginning on day 5 after birth, *Col2a1-CreER*^*TM*^*:Bmal1*^*flox/flox*^ mice were injected intraperitoneally with tamoxifen to knockout *Bmal1* in chondrocytes. Consistent with research mentioned before, *Col2a1-CreER*^*TM*^*:Bmal1*^*flox/flox*^ mice exhibited cartilage degradation and chondrocyte apoptosis, which deteriorated the condition of post-traumatic OA.^178^

### Others

Notably, bone/cartilage homeostasis is implicated with the condition of other systems, and *Bmal1*-deficiency in organs besides skeletal system do have influence on bone/cartilage formation. For example, models generated by Schroder et al. further revealed a relationship between intrinsic muscle clock, cartilage and bone. Derived from floxed *Bmal1* mouse and skeletal muscle-specific *Cre*-recombinase mouse (*HSA-Cre*), iMS*Bmal1*^*-/-*^ mice are solely deleted *Bmal1* in adult skeletal muscle. Consistent to the germline *Bmal1* knockout mice, significant bone and cartilage changes throughout the body were detected, which demonstrated declined joint collagen and elevated bone calcification, revealing that muscular clock is indispensable for musculoskeletal health.^82^

There are also studies suggesting crosstalk between digestive system and bone metabolism through employing conditional *Bmal1* knockout mice. Frank et al. indicated that specific deletion of *Bmal1* in colon epithelial cell of mice (*Ts4-Cre;Bmal1*^*fl/fl*^) could impair bone formation, which may be attributed to the disruption of gut microbiota and gut-derived hormones.^51^ Additionally, Kawai et al. generated a conditional knockout of *Bmal1* in the intestinal tissue (*Bmal1*_Int_^*-/-*^ mice) by crossing *Bmal1*^*fl/fl*^ mice with *Villin-Cre* mice homozygous for the floxed allele. The *Bmal1*_Int_^*-/-*^ mice also represented bone resorption due to impaired transcellular Ca^2+^ absorption and sympathetic activity, indicating that intestinal *Bmal1* is a primary system controlling the circadian of serum Ca^2+^ concentration.^179^

Additionally, multiple conditional *Bmal1* knockout mice were generated to investigate other phenotypes that are indirectly related to bone remodeling and cartilage metabolism. Huo et al. conditionally deleted *Bmal1* in myeloid cells and resulted in enhanced vascular remodeling.^180^ Hong et al. indicated that myeloid selective *Bmal1*-knockout mice (*Bmal1*^*f/f*^*, LysM*^*cre/+*^) led to exacerbated hypometabolic state and these mice were more sensitive to LPS treatment, which were prone to suffer endotoxemia. And their results suggested a role of *Bmal1* in attenuating NLRP3 inflammasome mediated inflammation.^181^ Although previous studies have proved that vascularization and inflammatory response had effects on bone/cartilage metabolism, further investigation is required to determine whether these conditional knockout mice exhibit changes in bone/cartilage metabolism.

As mentioned above, the phenotypes resulting from *Bmal1* knockout are not consistent across different mouse models. This may be attributed to (1) the non-specific expression of *Cre* recombinases, such as *Ctsk-Cre*, which was originally thought to be osteoclast-specific but recent studies have shown it can also be expressed in chondroprogenitor cells and osteoblast progenitors, affecting osteoclast and bone metabolism studies. (2) Rhythmic oscillations are inconsistent across different tissues and cells, and the expression and function of *Bmal1* are influenced by their specific physiological demands and environmental factors. (3) Peripheral clocks may communicate with each other. Through the reconstitution of *Bmal1* expression in liver in arrhythmic *Bmal1*-null mice, it is demonstrated the autonomy of the liver clock, and peripheral clocks are influenced by systemic signals from other clocks to maintain homeostasis.^182^ In conclusion, more research is needed to develop more specific *Bmal1* knockout models and to explore the specific roles of *Bmal1* in various biological processes.

## Conclusions and future perspectives

In this context, we summarize the potential downstream signaling pathway mechanisms and upstream control of *Bmal1* affecting bone/cartilage metabolism, as well as the used *Bmal1* knockout mouse models. Clearly, the current understanding of how exactly *Bmal1* (or circadian rhythm) is linked to bone/cartilage metabolism does not match the current knowledge of molecular details of bone/cartilage metabolism. Phenotypic studies are more often reported, and the phenotypes are not consistent across different cell lines, microenvironmental conditions and mouse models. Besides, the direct regulatory mechanism between *Bmal1* and these signaling pathways is unclear and controversial. These may be attributed to the cross-talk between various signaling pathways, and the involvement of various cytokines in forming a complex regulatory network. Aging, tissue and cell specificity, and crosstalk within peripheral clocks may also make a difference. More repeated experiments are needed for further verification.

Also, further research is needed to identify the target genes of *Bmal1* and elucidate the mechanisms by which *Bmal1* exerts its biological effects. For example, recently some studies have focused on *Bmal1*-regulated signaling axes related to bone metabolism, including the MTR1/AMPKβ1/BMAL1 signaling axis^156^ and the miR142-3p/BMAL1/YAP signaling axis^146^; some have cast their eyes on *Bmal1*-mediated regulation of oxidative stress pathways in macrophages to modulate inflammatory microenvironment for promising bone damage treatment^183^; and efforts are made to apply the knowledge to the field of tissue engineering to repair bone defects. These may be the future directions.

Under the control of circadian rhythms, the dynamic balance between bone formation and resorption ensures the integrity of bone mass and the stability of bone structure and function. Disruption in circadian rhythms, which leads to changes in the fate of BMSCs, is associated with the increased risks of osteoporosis, fractures, and osteoarthritis.^184,185^ *Bmal1* can be a promising target for the treatment and prevention of skeletal-related diseases. Further investigation on identifying efficient *Bmal1* regulators is also needed for the intervention of various bone/cartilage diseases.
